# Regional surveillance of medically-attended farm-related injuries in children and adolescents

**DOI:** 10.3389/fpubh.2022.1031618

**Published:** 2022-12-14

**Authors:** Jeffrey J. VanWormer, Richard L. Berg, Richard R. Burke, Kathrine L. Barnes, Bryan P. Weichelt

**Affiliations:** ^1^Center for Clinical Epidemiology and Population Health, Marshfield Clinic Research Institute, Marshfield, WI, United States; ^2^Office of Research Support Services, Marshfield Clinic Research Institute, Marshfield, WI, United States; ^3^National Farm Medicine Center, Marshfield Clinic Research Institute, Marshfield, WI, United States

**Keywords:** agriculture, injuries, children, surveillance, USA

## Abstract

**Purpose:**

Due to numerous environmental hazards such as heavy machinery and large livestock, youth who live and work on farms are at high risk of injury, disability, and death. This study described a regional surveillance system for monitoring farm-related injuries in children and adolescents. As the risk of farm-related injuries are not exclusive to farm residents, trends in farm-related injuries over the previous 5 years were reported and compared between children/adolescents who did and did not live on farms in north-central Wisconsin.

**Methods:**

A retrospective cohort of child and adolescent patients of the Marshfield Clinic Health System was assembled. Incident farm-related injuries, including from agricultural work or other activities in a farm environment, were extracted from medical records from 2017 through 2021. Generalized linear models were created to compare age- and sex-adjusted farm-related injury rates by year.

**Results:**

There were 4,730 (5%) in-farm and 93,420 (95%) out-farm children and adolescents in the cohort. There were 65 incident farm-related injury cases in the in-farm group and 412 in the out-farm group. The annual incidence rate of farm-related injuries was higher in the in-farm group, but changes during the 5-year timeframe were not significant in either group. In the in-farm group, rates ranged from a high of 61.8 [95% confidence interval (CI): 38.3, 94.5] incident farm-related injuries per 10,000 children/adolescents in 2017 to a low of 28.2 (13.5, 51.9) injuries per 10,000 children/adolescents in 2018. In the out-farm group, rates ranged from 10.7 (8.3, 13.6) to 16.8 (13.7, 20.5) incident farm-related injuries per 10,000 children/adolescents per year between 2017 and 2021. The in-farm group had a higher proportion of injured males and heavy machinery injuries, while the out-farm group had more all-terrain vehicle injuries and pesticide poisonings.

**Conclusion:**

Farm residency remains hazardous for children and adolescents, as injury rates were three times higher in the in-farm group and remained stable over 5 years. All-terrain vehicle injuries were high in both groups, and should be a priority in rural safety interventions. With additional adaptations to other states, this surveillance model could be scaled across other healthcare systems.

## Introduction

At approximately 23 fatal injuries per 100,000 workers per year in the U.S., agriculture, forestry, and fishing consistently have the highest rates of occupational fatalities of any industry sector (1). Fatalities also occur in children and adolescents who live and work on farms (2, 3). Estimates are dated, but 893,000 youth under age 20 lived on farms as of 2014, with roughly half performing work duties and an additional 260,000 youth farm employees, meaning over 1 million U.S. children and adolescents have at least some regular level of exposure to farm hazards (4).

Farm family members live in a hazardous environment that is focused on agricultural production, thus the burden of injuries is prodigious (5). Young workers (regardless of whether they live on a farm or not) are eight times more likely to die from farm work as compared to all other industries combined, and over half of youth injured on a farm were not actually working at the time of their injury (6). All-terrain vehicles (ATVs), for example, are a primary cause of farm-related injuries in children and adolescents (7), and an estimated 12% of farm-related injuries appearing in news media reports are due to heavy machinery such as skid steers or augers (8).

Little is known about recent trends in farm-related injury risks, however, because the U.S. lacks a comprehensive surveillance system. National farm injury estimates are particularly limited after 2015 when federal occupational health agencies suspended farm-related injury surveillance activities. There may be some positive developments, as the absolute number of pediatric visits to emergency rooms for agriculture-related injuries in the U.S. has declined by over 40% since 2001 (9, 10). This seeming improvement may be driven by the declining numbers of farm households though, as fewer youth actually live or work on farms in the U.S. today (11, 12). As such, youth's relative risk for farm-related injuries remains unclear. This underscores the critical need for reliable, simultaneous capture of both injuries and time at risk when assessing farm-related injury trends, particularly for priority populations like children and adolescents (13). Leaders of agricultural safety organizations have identified farm-related injury surveillance as a top priority to help evaluate safety initiatives (2, 14–16).

The burden of farm-related injury is not exclusive to youth who live on farms. Such injuries, perhaps about one-third, have also been shown to occur in adolescents who work on a farm or in young children playing on a friend's or family's farm (17, 18). For example, nearly 25 million youth visit farms in the U.S. annually, with nearly all of them being repeat visitors (19). As might be expected, one study in Alberta, Canada observed the relative risk of farm-related injury was far greater in children and adolescents who live on rural farms as compared to their urban non-farm counterparts (20). Such estimates are not available in the U.S. though. Furthermore, there could be differences in risk depending on the specific type of farm-related injury. Studies with direct demographic comparisons across defined populations are rare though, and farm-related injury risk estimates from the U.S. are dated (19). The purpose of this study was to: (1) describe the Wisconsin National Children's Center for Rural and Agricultural Health and Safety surveillance (WINS) system for regional monitoring of farm-related injuries in children and adolescents, (2) estimate annualized trends in farm-related injuries over the previous 5 years, and (3) compare farm-related injury trends between children/adolescents who did vs. did not live on area farms.

## Methods

### Design and setting

**Figure 1:**
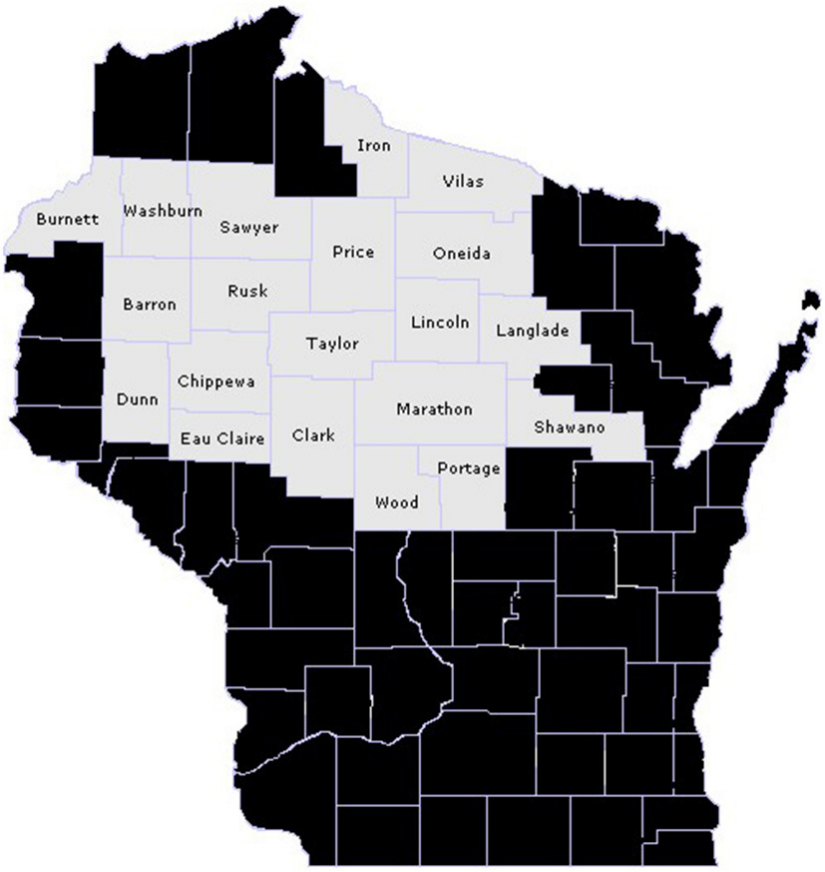
20-county target population of north-central Wisconsin children and adolescents who did and did not live on a farm in 2017–2021.

The source population included child and adolescent patients of the Marshfield Clinic Health System (MCHS) who lived in a 20-county region of north-central Wisconsin during the 2017–2021 timeframe (Figure 1), and who had reasonably complete capture of their medical care within MCHS data systems. MCHS is a large multispecialty system that serves a predominantly rural patient base across small communities in central and northern Wisconsin. As detailed further below, study data was extracted from MCHS's research data repository, which stores medical and administrative information documented in MCHS electronic health records (EHR) from clinical encounters.

### Cohort assembly

The analytical cohort included children and adolescents in the source population who: (1) were age 0–17 years for ≥90 continuous days between 01/01/2017 and 12/31/2021, and (2) had reasonably complete capture of medical care within MCHS data systems as evidenced by: (a) “medically homed” to an MCHS medical center (i.e., ≥ 2 preventive or well-child visits over the previous 3 years or an assigned MCHS primary care provider), (b) member of the MCHS-affiliated Security Health Plan (SHP) of Wisconsin, or (c) resident of the Marshfield Epidemiologic Study Area (MESA) (21, 22). Person-time follow-up, or cohort entry, for individuals began on the first day of the month that all eligibility criteria were met within the study timeframe. Cohort end was due to death, out-migration, or reaching age 18 years.

### Farm status

Cohort members were stratified by farm and non-farm residency. Children and adolescents with a residential address that had evidence of agricultural production at any point during the 2017–2021 study timeframe were categorized in the in-farm group. Those with no evidence of farm residence during that timeframe were categorized in the out-farm group. To establish farm residency, cohort members' MCHS residential address history was linked to two separate publicly-available farm data sources, including (1) a registry of licensed dairy producers from Wisconsin's Department of Agriculture, Trade and Consumer Protection, or (2) a commercially-available (purchased) listing of agricultural producers (www.dtn.com/agriculture/producer/) specific to the target 20-county region. All study procedures were approved by the MCHS Institutional Review Board, including approvals to waive documentation of informed consent and HIPAA authorization.

### Farm-related injuries

The outcome was medically-attended farm-related injuries. A farm-related injury was operationally defined as medical attention received for an energy transfer event that originated from a farm source (e.g., tractor, tool, livestock) while the individual was either in a farm location or engaged in (or observing) farm-related work. Farm-related injuries could be agricultural work or other activities in a farm environment. Efforts were also made to identify recreational activities that took place in the farm location (e.g., riding horses or ATVs). Medically-attended farm-related injuries were ascertained based on adaptations of injury surveillance models outlined by Landsteiner et al. (23) and Scott et al. (24, 25). Injury details were extracted from medical diagnoses observed during emergency, inpatient, urgent care, or outpatient encounters in the MCHS electronic data repository. Said diagnoses typically occurred in MCHS-affiliated hospitals and clinics, but were also ascertained from SHP insurance claims for encounters outside of MCHS (for those with SHP insurance). Specifically, external cause of injury codes indicative of farm accidents have been shown to capture farm-related injuries with excellent specificity (26, 27) and their use has been mandated in medical coding practices in Wisconsin since 1993 (28). Such codes are primarily used for billing as part of the 10th revision of the International Statistical Classification of Diseases and Related Health Problems (ICD-10) system. These codes included Y92.7X and W30.XXX, in addition to several less specific codes (26, 29) that are described in the Appendix in Supplementary material.

### Chart review

In the in-farm group, all farm-related injuries identified *via* the electronic case finding logic were subjected to a manual chart review conducted by a trained Research Coordinator. This chart audit included a review of all clinical documentation of the associated medical encounter in order to confirm the presence or absence of a farm-related injury. The Research Coordinator primarily reviewed free-text documents to determine whether the injury occurred while doing or observing a farm activity, related or unrelated recreational activity, or otherwise at a farm location. Chart audits further extracted clinical features of the farm-related injury and documented precipitating circumstances. Approximately 10% of all candidate cases were re-audited by a different Research Coordinator to detect and correct possible interrater discrepancies or inconsistencies. Injury chart reviews have not yet been conducted in the out-farm group due to study budget constraints (thus detailed injury features were only available in the in-farm group), but chart reviews will be done in both groups as part of future farm-related injury surveillance activities.

### Analyses

Descriptive characteristics were reported on the farm and out-farm groups. Differences between injured individuals in the farm vs. out-farm group were also summarized. For the injury trends analysis, new injury episodes were required to be separated by ≥30 days and age- and sex-adjusted incidence rates, with robust 95% confidence intervals (CI), were reported using the direct standardization method based on the 2010 Wisconsin Census population. Incident annual injury rates were modeled using generalized linear mixed models. Analytical procedures were conducted using SAS version 9.4 (Cary, NC).

## Results

There were 98,150 unique children and adolescents in the WINS cohort, including 4,730 (5%) in the farm and 93,420 (95%) in the out-farm group. As outlined in Table 1, the two groups were relatively similar across most sociodemographic characteristics, but the in-farm group was slightly older, had more White, non-Hispanic children/adolescents, and fewer with public-assisted health insurance. Across the 2017–2021 timeframe, mean ± SD months of available follow-up (i.e., time under observation) was generally high, and somewhat greater in the in-farm group (43.5 ± 1 8.0 farm vs. 39.2 ± 18.9 non-farm). Annualized person-year contributions were relatively stable in both groups, varying < 10% between years and ranging from a high of 3,554 person-years in the in-farm group and 63,103 person-years in the out-farm group in 2019, to a low of 3,251 person-years in the in-farm group and 58,860 person-years in the out-farm group in 2021.

**Table 1:** Characteristics of children and adolescents in north-central Wisconsin who did and did not live on a farm in 2017–2021.

	**In-farm**	**Out-farm**
	***n* = 4,730**	***n* = 93,420**
Age (yrs, at cohort end)	12.4 ± 6.1	11.6 ± 5.9
Gender		
Female	2,251 (48%)	45,898 (49%)
Male	2,479 (52%)	47,522 (51%)
Race/Ethnicity		
White, non-Hispanic	4,031 (85%)	72,470 (78%)
Non-White or Hispanic	305 (6%)	13,955 (15%)
Unknown	394 (8%)	6,995 (7%)
Health insurance		
Private	2,353 (50%)	42,609 (46%)
Public-assisted	1,973 (42%)	44,822 (48%)
None	404 (9%)	5,989 (6%)
Number of ambulatory visits (over 5 years)	10.3 ± 13.0	10.2 ± 12.2
Body mass index (percentile categories)		
Obese	786 (17%)	16,370 (18%)
Overweight	699 (15%)	13,625 (15%)
Normal weight	2,521 (53%)	48,923 (52%)
Underweight	199 (4%)	4,637 (5%)
Unknown	525 (11%)	9,865 (11%)
Current smoker	61 (2%)	1,815 (3%)
Chronic medical condition	1,145 (24%)	24,908 (27%)

Values are reported as mean ± SD or frequency (% of total).

There were 69 farm-related injuries in the in-farm group [65 (1.4%) unique individuals] and 412 in the out-farm group [378 (0.4%) unique individuals] during 2017–2021. Among the injuries in the in-farm group, all but two were confirmed upon manual chart review. Both unconfirmed farm-related injuries were miscoded as involving recreational vehicles, one having occurred completely outside of the farm environment or during farming activities, and the other not actually involving a recreational vehicle. Inter-rater agreement with chart re-reviews was 100%. Basic characteristics of the incident farm-related injuries are outlined in Table 2, stratified by the in-farm vs. out-farm group. The in-farm group had a higher proportion of injured males. The out-farm group had a different composition of injury types, with more ATV and fewer farm machinery injuries. Most farm-related injuries occurred during summer and in adolescents. Other injury features were only available from chart audits in the in-farm group. The most common injury location was lower extremities (38%), followed by upper extremities (26%), head (22%), and torso (14%). There were no known fatalities linked to patients' farm-related injuries. The vast majority of patients in the in-farm group initially presented to a hospital emergency room (94%). In terms of injury antecedents, 33% were attributed to play or recreation, while 26% were attributed to farm work, and 16% were attributed to transportation. The remainder were unclear from medical chart notes.

**Table 2:** Features of incident farm-related injuries in north-central Wisconsin children and adolescents in 2017–2021, stratified by those who did vs. did not live on a farm.

	**In-farm**	**Out-farm**
	***n* = 65**	***n* = 378**
Age (yrs, at time of injury)	12.0 ± 4.2	12.4 ± 4.4
0–5	9 (14%)	43 (11%)
6–11	20 (31%)	105 (28%)
12–17	36 (55%)	230 (61%)
Gender		
Female	25 (38%)	181 (48%)
Male	40 (62%)	197 (52%)
Season		
Spring	6 (9%)	80 (21%)
Summer	32 (49%)	173 (45%)
Fall	16 (25%)	86 (23%)
Winter	11 (17%)	39 (10%)
Injury diagnosis (external cause)		
Farm-unspecified	18 (28%)	39 (10%)
All-terrain vehicle	17 (26%)	182 (48%)
Animal strike	14 (22%)	98 (26%)
Animal riding	9 (14%)	50 (13%)
Farm machinery	7 (11%)	2 (1%)
Pesticide/herbicide poisoning	0 (0%)	5 (1%)
Other	0 (0%)	2 (1%)
Injury areas		
Lower extremities	25 (38%)	
Upper extremities	17 (26%)	
Head	14 (22%)	NA
Torso	9 (14%)	
Spine/neck	0 (0%)	

Values are reported as mean ±SD or frequency (% of total).

**Figure 2:**
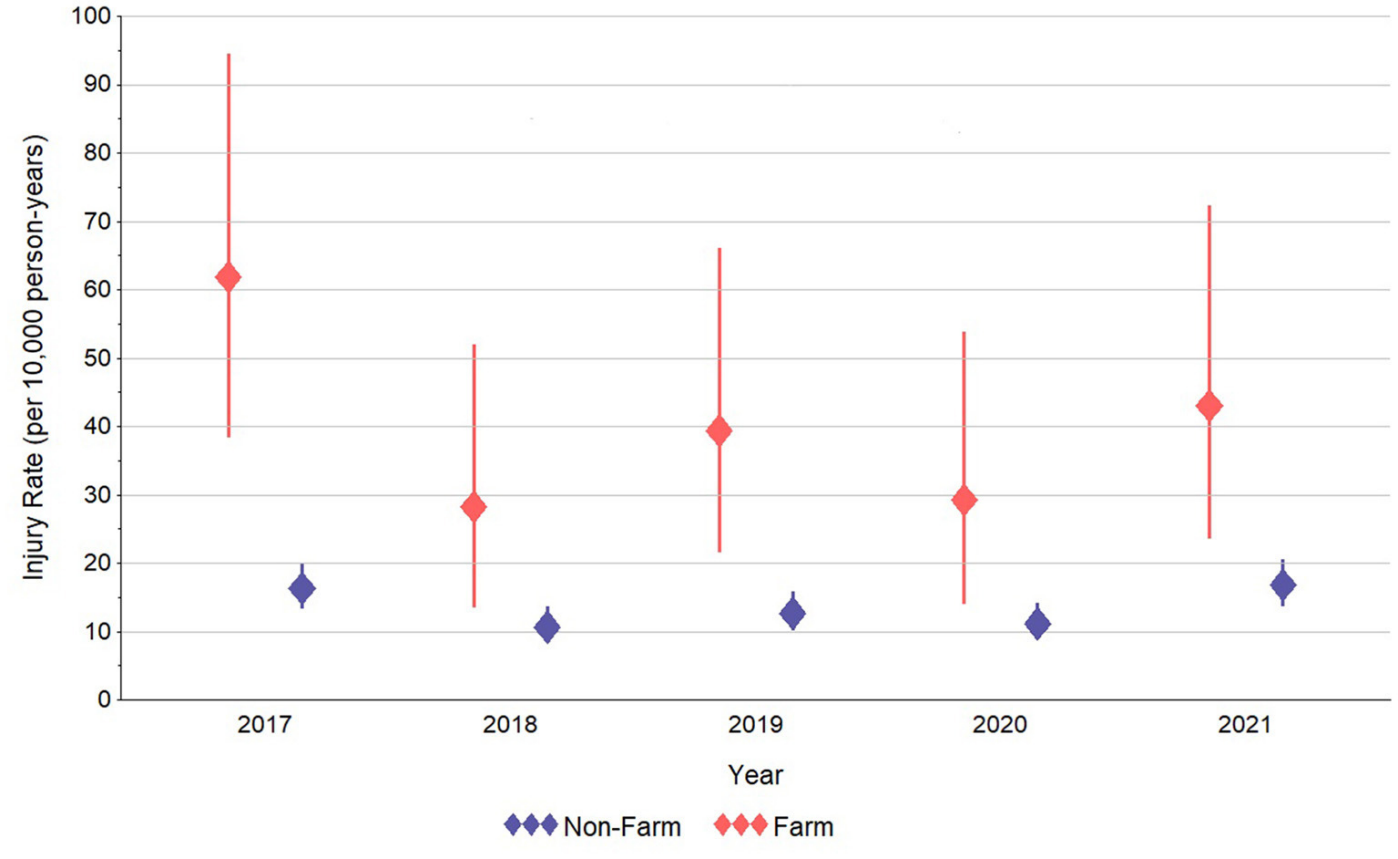
Estimated annual incidence rate of farm-related injuries in north-central Wisconsin children and adolescents who did and did not live on a farm in 2017–2021.

As outlined in Figure 2, the annual incidence rate of farm-related injuries was clearly higher in the in-farm group. Changes during the 5-year study timeframe were not significant in either group, but were very steady in the out-farm group, ranging from 10.7 (95% confidence interval [CI]: 8.3, 13.6) to 16.8 (CI: 13.7, 20.5) incident farm-related injuries per 10,000 children/adolescents per year between 2017 and 2021. In the in-farm group, rates ranged from a high of 61.8 (CI: 38.3, 94.5) incident farm-related injuries per 10,000 children/adolescents in 2017 to a low of 28.2 (CI: 13.5, 51.9) injuries per 10,000 children/adolescents in 2018. Incident injury rates were generally stable in the in-farm group since 2018.

## Discussion

The early intent of WINS was to establish a (largely) passive farm-related surveillance methodology in children and adolescents. While limited in scope to north-central Wisconsin, we were able to link available data on farm residency to the medical records of child/adolescent patients of the major healthcare provider in the region. This permitted the subsequent examination of farm-related injuries in the WINS cohort. Results indicated that, over the past 5 years, approximately 40 per 10,000 children/adolescents who lived on farms in north-central Wisconsin experienced a farm-related injury. Though modestly lower since the high point in 2017, incident farm-related injury rates in the in-farm group were statistically indistinguishable between years. This relatively “flat” trend in farm-related injuries was similarly observed in the out-farm group, though, as expected based on prior studies indicating the burden of farm-related injuries is highest in children of farm owners (2, 17, 19), injury rates in the in-farm group were typically three times that of the out-farm group.

Recent data on injury rates in U.S. farm children are sparse, but the risk of farm-related injury in WINS was slightly lower than observed in Alberta, Canada in the early 2000's (20). WINS injury rates were also somewhat lower than the most recent available national estimates of farm-related injury risks in U.S. youth from Hendricks et al. (19). The unit of analysis in that study was farm (vs. farm residents) and injuries were reported by household members, thus some caution is warranted regarding direct comparisons to prior studies with different methodologies. However, under an assumption of 1.5 children and adolescents per farm residence in our target region of north-central Wisconsin (30), the Hendricks, et al. study (based on 2001–2014 surveys) would predict an annualized risk of self-reported farm-related injuries of ~0.6% in our in-farm group, whereas we observed an actual annualized risk of medically-attended farm-related injuries of 0.4%.

Injuries in our study were more common during summer months, as observed by others (6, 23). Injuries were also more common in adolescents, who presumably had more exposure to agricultural work and/or hazards. Serious injuries to the upper and lower extremities that presented in the emergency room were common, but no fatalities were observed in this dataset. Injury causes clearly differed between the in-farm and out-farm groups. Consistent with prior national estimates (6), farm-related injuries for in-farm children were more balanced across ATVs, animal strikes, and other farm causes. Out-farm children, however, were rarely injured by farm machinery, but were more likely to have experienced an ATV injury. Farming environments are known to be major hazards for children and adolescents in regard to motorized accidents (31), but our findings may also reflect the recent proliferation of non-occupational, recreational ATV use across the U.S. (32). This highlights the need for more effective ATV operator safety training for all youth, and perhaps more restrictive requirements (or enforcement) of ATV operation in those who have not completed safety training. Also of note, pesticide/herbicide poisonings were only observed in the out-farm group, which differed from prior findings by Kim et al. (20). This could indicate increasingly cautious application practices in farm families that are more familiar and experienced with chemical hazards. More applicator training on pesticide exposure prevention may be needed in the general population, including broader efforts to minimize pesticide use and reduce chemical drift (33).

### Strengths and limitations

Strengths of WINS included the objective EHR and claims outcomes, as well as the population-based sample that reflected real-world patterns of farm-related injuries in north-central Wisconsin children and adolescents. The chief limitation was the retrospective use of medical records to identify injury cases. Individuals with minor injuries who did not receive medical care were not captured, thus incidence rates in this study are likely conservative and err toward more severe accidents. In addition, the target population had a limited regional scope that may be somewhat over-representative of dairy production relative to other parts of the U.S. The in-farm group was based solely on residency, assuming all individuals who lived on an active farm had approximately equal risk. While this assumption was perhaps reasonable given that most Wisconsin farms are still family owned and operated (34), our design did not permit more detailed permutations of risk, such as classifying those who spend more time doing specific dangerous activities, or parsing the out-farm group into those who worked on or regularly visited a farm. Future research could consider more detailed validation of in-farm and out-farm residency in order to help quantify degrees of exposure to agricultural hazards by other related factors such as acreage dedicated to production, volume of heavy machinery, specific farm types (e.g., dairy, crop types), and time spent in farm-related activities, including work and recreation.

## Conclusions

Future iterations of the WINS platform will begin integrating other data sources from the state of Wisconsin [e.g., ambulance runs based on models outlined by Scott et al. (24, 25)] to capture more injuries that may have been treated outside of MCHS or not covered by SHP insurance. In addition, chart reviews will be conducted on a sample of farm-related injuries in the out-farm group, and the WINS source population will be expanded by including other healthcare systems that serve children and adolescents beyond north-central regions of Wisconsin. An expanded population will permit identification of subgroups where injury trends may be more or less favorable (35). In addition, the WINS model can be leveraged for other studies designed to examine etiologies of farm-related injuries, or other health and safety concerns, in farm children and adolescents.

Direct calls and intimations for improved farm-related injury surveillance in the U.S. are widespread (2, 12–16). Relative to prior studies on this topic, which typically relied on aggregated estimates of the at-risk population, WINS was able to track and compare injuries in a defined cohort of children and adolescents who did and did not live on active farms, and where the majority of their medical care was captured. The informatics methods used in this injury surveillance model, which combines data from both private healthcare and publicly available sources, are fairly practical and can be scaled more broadly across other healthcare systems in rural areas with large populations of farm or ranch families. With additional data, this regional project can serve as an initial step toward developing an integrated national farm-related injury surveillance network, which will help farm safety advocates create and track near-term priorities for prevention.

## Data availability statement

The datasets for this study are unavailable for public access because informed consent to share said data (beyond the research team) was not obtained from study participants, but are available from the corresponding author on reasonable request.

## Ethics statement

The studies involving human participants were reviewed and approved by Marshfield Clinic Research Institute. Written informed consent from the participants' legal guardian/next of kin was not required to participate in this study in accordance with the National Legislation and the Institutional Requirements.

## Author contributions

JV was the study PI and led the methodological design and execution, as well as drafted the initial manuscript. RLB led statistical analyses and critically reviewed manuscript draft. RRB led data collection activities and critically reviewed manuscript drafts. KB managed the study, assisted with data collection activities, and critically reviewed manuscript drafts. BW assisted with the methodological design and critically reviewed manuscript drafts. All authors contributed to the article and approved the submitted version.
