# Supplementary material for: Regional surveillance of medically-attended farm-related injuries in children and adolescents
Source: Front Public Health. 2022 Dec 14;10:1031618. doi: 10.3389/fpubh.2022.1031618 (PMC9795044; doi:10.3389/fpubh.2022.1031618)
Supplement: Supplementary file 1 [file Table_1.DOCX]

Appendix. Diagnosis codes to identify agricultural injuries.

| **ICD-10 code** | **Description** |
| --- | --- |
| T60.0X1 | Toxic effect of organophosphate and carbamate insecticides, accidental (unintentional) |
| T60.0X1A | Toxic effect of organophosphate and carbamate insecticides, accidental (unintentional), initial encounter |
| T60.0X1D | Toxic effect of organophosphate and carbamate insecticides, accidental (unintentional), subsequent encounter |
| T60.0X1S | Toxic effect of organophosphate and carbamate insecticides, accidental (unintentional), sequela |
| T60.0X4 | Toxic effect of organophosphate and carbamate insecticides, undetermined |
| T60.0X4A | Toxic effect of organophosphate and carbamate insecticides, undetermined, initial encounter |
| T60.0X4D | Toxic effect of organophosphate and carbamate insecticides, undetermined, subsequent encounter |
| T60.0X4S | Toxic effect of organophosphate and carbamate insecticides, undetermined, sequela |
| T60.2X1 | Toxic effect of other insecticides, accidental (unintentional) |
| T60.2X1A | Toxic effect of other insecticides, accidental (unintentional), initial encounter |
| T60.2X1D | Toxic effect of other insecticides, accidental (unintentional), subsequent encounter |
| T60.2X1S | Toxic effect of other insecticides, accidental (unintentional), sequela |
| T60.2X4 | Toxic effect of other insecticides, undetermined |
| T60.2X4A | Toxic effect of other insecticides, undetermined, initial encounter |
| T60.2X4D | Toxic effect of other insecticides, undetermined, subsequent encounter |
| T60.2X4S | Toxic effect of other insecticides, undetermined, sequela |
| T60.3X1 | Toxic effect of herbicides and fungicides, accidental (unintentional) |
| T60.3X1A | Toxic effect of herbicides and fungicides, accidental (unintentional), initial encounter |
| T60.3X1D | Toxic effect of herbicides and fungicides, accidental (unintentional), subsequent encounter |
| T60.3X1S | Toxic effect of herbicides and fungicides, accidental (unintentional), sequela |
| T60.3X4 | Toxic effect of herbicides and fungicides, undetermined |
| T60.3X4A | Toxic effect of herbicides and fungicides, undetermined, initial encounter |
| T60.3X4D | Toxic effect of herbicides and fungicides, undetermined, subsequent encounter |
| T60.3X4S | Toxic effect of herbicides and fungicides, undetermined, sequela |
| T60.4X1 | Toxic effect of rodenticides, accidental (unintentional) |
| T60.4X1A | Toxic effect of rodenticides, accidental (unintentional), initial encounter |
| T60.4X1D | Toxic effect of rodenticides, accidental (unintentional), subsequent encounter |
| T60.4X1S | Toxic effect of rodenticides, accidental (unintentional), sequela |
| T60.4X4 | Toxic effect of rodenticides, undetermined |
| T60.4X4A | Toxic effect of rodenticides, undetermined, initial encounter |
| T60.4X4D | Toxic effect of rodenticides, undetermined, subsequent encounter |
| T60.4X4S | Toxic effect of rodenticides, undetermined, sequela |
| T60.8X1 | Toxic effect of other pesticides, accidental (unintentional) |
| T60.8X1A | Toxic effect of other pesticides, accidental (unintentional), initial encounter |
| T60.8X1D | Toxic effect of other pesticides, accidental (unintentional), subsequent encounter |
| T60.8X1S | Toxic effect of other pesticides, accidental (unintentional), sequela |
| T60.8X4 | Toxic effect of other pesticides, undetermined |
| T60.8X4A | Toxic effect of other pesticides, undetermined, initial encounter |
| T60.8X4D | Toxic effect of other pesticides, undetermined, subsequent encounter |
| T60.8X4S | Toxic effect of other pesticides, undetermined, sequela |
| T60.91XA | Toxic effect of unspecified pesticide, accidental (unintentional), initial encounter |
| T60.91XD | Toxic effect of unspecified pesticide, accidental (unintentional), subsequent encounter |
| T60.91XS | Toxic effect of unspecified pesticide, accidental (unintentional), sequela |
| T60.94XA | Toxic effect of unspecified pesticide, undetermined, initial encounter |
| T60.94XD | Toxic effect of unspecified pesticide, undetermined, subsequent encounter |
| T60.94XS | Toxic effect of unspecified pesticide, undetermined, sequela |
| V09.1 | Pedestrian injured in unspecified nontraffic accident |
| V09.1XXA | Pedestrian injured in unspecified nontraffic accident, initial encounter |
| V09.1XXD | Pedestrian injured in unspecified nontraffic accident, subsequent encounter |
| V09.1XXS | Pedestrian injured in unspecified nontraffic accident, sequela |
| V80.0 | Animal-rider or occupant of animal drawn vehicle injured by fall from or being thrown from animal or animal-drawn vehicle in noncollision accident |
| V80.01 | Animal-rider injured by fall from or being thrown from animal in noncollision accident |
| V80.010 | Animal-rider injured by fall from or being thrown from horse in noncollision accident |
| V80.010A | Animal-rider injured by fall from or being thrown from horse in noncollision accident, initial encounter |
| V80.010D | Animal-rider injured by fall from or being thrown from horse in noncollision accident, subsequent encounter |
| V80.010S | Animal-rider injured by fall from or being thrown from horse in noncollision accident, sequela |
| V80.018 | Animal-rider injured by fall from or being thrown from other animal in noncollision accident |
| V80.018A | Animal-rider injured by fall from or being thrown from other animal in noncollision accident, initial encounter |
| V80.018D | Animal-rider injured by fall from or being thrown from other animal in noncollision accident, subsequent encounter |
| V80.018S | Animal-rider injured by fall from or being thrown from other animal in noncollision accident, sequela |
| V80.02 | Occupant of animal-drawn vehicle injured by fall from or being thrown from animal-drawn vehicle in noncollision accident |
| V80.02XA | Occupant of animal-drawn vehicle injured by fall from or being thrown from animal-drawn vehicle in noncollision accident, initial encounter |
| V80.02XD | Occupant of animal-drawn vehicle injured by fall from or being thrown from animal-drawn vehicle in noncollision accident, subsequent encounter |
| V80.02XS | Occupant of animal-drawn vehicle injured by fall from or being thrown from animal-drawn vehicle in noncollision accident, sequela |
| V80.1 | Animal-rider or occupant of animal-drawn vehicle injured in collision with pedestrian or animal |
| V80.11 | Animal-rider injured in collision with pedestrian or animal |
| V80.11XA | Animal-rider injured in collision with pedestrian or animal, initial encounter |
| V80.11XD | Animal-rider injured in collision with pedestrian or animal, subsequent encounter |
| V80.11XS | Animal-rider injured in collision with pedestrian or animal, sequela |
| V80.12 | Occupant of animal-drawn vehicle injured in collision with pedestrian or animal |
| V80.12XA | Occupant of animal-drawn vehicle injured in collision with pedestrian or animal, initial encounter |
| V80.12XD | Occupant of animal-drawn vehicle injured in collision with pedestrian or animal, subsequent encounter |
| V80.12XS | Occupant of animal-drawn vehicle injured in collision with pedestrian or animal, sequela |
| V80.2 | Animal-rider or occupant of animal-drawn vehicle injured in collision with pedal cycle |
| V80.21 | Animal-rider injured in collision with pedal cycle |
| V80.21XA | Animal-rider injured in collision with pedal cycle, initial encounter |
| V80.21XD | Animal-rider injured in collision with pedal cycle, subsequent encounter |
| V80.21XS | Animal-rider injured in collision with pedal cycle, sequela |
| V80.22 | Occupant of animal-drawn vehicle injured in collision with pedal cycle |
| V80.22XA | Occupant of animal-drawn vehicle injured in collision with pedal cycle, initial encounter |
| V80.22XD | Occupant of animal-drawn vehicle injured in collision with pedal cycle, subsequent encounter |
| V80.22XS | Occupant of animal-drawn vehicle injured in collision with pedal cycle, sequela |
| V80.3 | Animal-rider or occupant of animal-drawn vehicle injured in collision with two- or three-wheeled motor vehicle |
| V80.31 | Animal-rider injured in collision with two- or three-wheeled motor vehicle |
| V80.31XA | Animal-rider injured in collision with two- or three-wheeled motor vehicle, initial encounter |
| V80.31XD | Animal-rider injured in collision with two- or three-wheeled motor vehicle, subsequent encounter |
| V80.31XS | Animal-rider injured in collision with two- or three-wheeled motor vehicle, sequela |
| V80.32 | Occupant of animal-drawn vehicle injured in collision with two- or three-wheeled motor vehicle |
| V80.32XA | Occupant of animal-drawn vehicle injured in collision with two- or three-wheeled motor vehicle, initial encounter |
| V80.32XD | Occupant of animal-drawn vehicle injured in collision with two- or three-wheeled motor vehicle, subsequent encounter |
| V80.32XS | Occupant of animal-drawn vehicle injured in collision with two- or three-wheeled motor vehicle, sequela |
| V80.4 | Animal-rider or occupant of animal-drawn vehicle injured in collision with car, pick-up truck, van, heavy transport vehicle or bus |
| V80.41 | Animal-rider injured in collision with car, pick-up truck, van, heavy transport vehicle or bus |
| V80.41XA | Animal-rider injured in collision with car, pick-up truck, van, heavy transport vehicle or bus, initial encounter |
| V80.41XD | Animal-rider injured in collision with car, pick-up truck, van, heavy transport vehicle or bus, subsequent encounter |
| V80.41XS | Animal-rider injured in collision with car, pick-up truck, van, heavy transport vehicle or bus, sequela |
| V80.42 | Occupant of animal-drawn vehicle injured in collision with car, pick-up truck, van, heavy transport vehicle or bus |
| V80.42XA | Occupant of animal-drawn vehicle injured in collision with car, pick-up truck, van, heavy transport vehicle or bus, initial encounter |
| V80.42XD | Occupant of animal-drawn vehicle injured in collision with car, pick-up truck, van, heavy transport vehicle or bus, subsequent encounter |
| V80.42XS | Occupant of animal-drawn vehicle injured in collision with car, pick-up truck, van, heavy transport vehicle or bus, sequela |
| V80.5 | Animal-rider or occupant of animal-drawn vehicle injured in collision with other specified motor vehicle |
| V80.51 | Animal-rider injured in collision with other specified motor vehicle |
| V80.51XA | Animal-rider injured in collision with other specified motor vehicle, initial encounter |
| V80.51XD | Animal-rider injured in collision with other specified motor vehicle, subsequent encounter |
| V80.51XS | Animal-rider injured in collision with other specified motor vehicle, sequela |
| V80.52 | Occupant of animal-drawn vehicle injured in collision with other specified motor vehicle |
| V80.52XA | Occupant of animal-drawn vehicle injured in collision with other specified motor vehicle, initial encounter |
| V80.52XD | Occupant of animal-drawn vehicle injured in collision with other specified motor vehicle, subsequent encounter |
| V80.52XS | Occupant of animal-drawn vehicle injured in collision with other specified motor vehicle, sequela |
| V80.6 | Animal-rider or occupant of animal-drawn vehicle injured in collision with railway train or railway vehicle |
| V80.61 | Animal-rider injured in collision with railway train or railway vehicle |
| V80.61XA | Animal-rider injured in collision with railway train or railway vehicle, initial encounter |
| V80.61XD | Animal-rider injured in collision with railway train or railway vehicle, subsequent encounter |
| V80.61XS | Animal-rider injured in collision with railway train or railway vehicle, sequela |
| V80.62 | Occupant of animal-drawn vehicle injured in collision with railway train or railway vehicle |
| V80.62XA | Occupant of animal-drawn vehicle injured in collision with railway train or railway vehicle, initial encounter |
| V80.62XD | Occupant of animal-drawn vehicle injured in collision with railway train or railway vehicle, subsequent encounter |
| V80.62XS | Occupant of animal-drawn vehicle injured in collision with railway train or railway vehicle, sequela |
| V80.7 | Animal-rider or occupant of animal-drawn vehicle injured in collision with other nonmotor vehicles |
| V80.71 | Animal-rider or occupant of animal-drawn vehicle injured in collision with animal being ridden |
| V80.710 | Animal-rider injured in collision with other animal being ridden |
| V80.710A | Animal-rider injured in collision with other animal being ridden, initial encounter |
| V80.710D | Animal-rider injured in collision with other animal being ridden, subsequent encounter |
| V80.710S | Animal-rider injured in collision with other animal being ridden, sequela |
| V80.711 | Occupant of animal-drawn vehicle injured in collision with animal being ridden |
| V80.711A | Occupant of animal-drawn vehicle injured in collision with animal being ridden, initial encounter |
| V80.711D | Occupant of animal-drawn vehicle injured in collision with animal being ridden, subsequent encounter |
| V80.711S | Occupant of animal-drawn vehicle injured in collision with animal being ridden, sequela |
| V80.72 | Animal-rider or occupant of animal-drawn vehicle injured in collision with other animal-drawn vehicle |
| V80.720 | Animal-rider injured in collision with animal-drawn vehicle |
| V80.720A | Animal-rider injured in collision with animal-drawn vehicle, initial encounter |
| V80.720D | Animal-rider injured in collision with animal-drawn vehicle, subsequent encounter |
| V80.720S | Animal-rider injured in collision with animal-drawn vehicle, sequela |
| V80.721 | Occupant of animal-drawn vehicle injured in collision with other animal-drawn vehicle |
| V80.721A | Occupant of animal-drawn vehicle injured in collision with other animal-drawn vehicle, initial encounter |
| V80.721D | Occupant of animal-drawn vehicle injured in collision with other animal-drawn vehicle, subsequent encounter |
| V80.721S | Occupant of animal-drawn vehicle injured in collision with other animal-drawn vehicle, sequela |
| V80.73 | Animal-rider or occupant of animal-drawn vehicle injured in collision with streetcar |
| V80.730 | Animal-rider injured in collision with streetcar |
| V80.730A | Animal-rider injured in collision with streetcar, initial encounter |
| V80.730D | Animal-rider injured in collision with streetcar, subsequent encounter |
| V80.730S | Animal-rider injured in collision with streetcar, sequela |
| V80.731 | Occupant of animal-drawn vehicle injured in collision with streetcar |
| V80.731A | Occupant of animal-drawn vehicle injured in collision with streetcar, initial encounter |
| V80.731D | Occupant of animal-drawn vehicle injured in collision with streetcar, subsequent encounter |
| V80.731S | Occupant of animal-drawn vehicle injured in collision with streetcar, sequela |
| V80.79 | Animal-rider or occupant of animal-drawn vehicle injured in collision with other nonmotor vehicles |
| V80.790 | Animal-rider injured in collision with other nonmotor vehicles |
| V80.790A | Animal-rider injured in collision with other nonmotor vehicles, initial encounter |
| V80.790D | Animal-rider injured in collision with other nonmotor vehicles, subsequent encounter |
| V80.790S | Animal-rider injured in collision with other nonmotor vehicles, sequela |
| V80.791 | Occupant of animal-drawn vehicle injured in collision with other nonmotor vehicles |
| V80.791A | Occupant of animal-drawn vehicle injured in collision with other nonmotor vehicles, initial encounter |
| V80.791D | Occupant of animal-drawn vehicle injured in collision with other nonmotor vehicles, subsequent encounter |
| V80.791S | Occupant of animal-drawn vehicle injured in collision with other nonmotor vehicles, sequela |
| V80.8 | Animal-rider or occupant of animal-drawn vehicle injured in collision with fixed or stationary object |
| V80.81 | Animal-rider injured in collision with fixed or stationary object |
| V80.81XA | Animal-rider injured in collision with fixed or stationary object, initial encounter |
| V80.81XD | Animal-rider injured in collision with fixed or stationary object, subsequent encounter |
| V80.81XS | Animal-rider injured in collision with fixed or stationary object, sequela |
| V80.82 | Occupant of animal-drawn vehicle injured in collision with fixed or stationary object |
| V80.82XA | Occupant of animal-drawn vehicle injured in collision with fixed or stationary object, initial encounter |
| V80.82XD | Occupant of animal-drawn vehicle injured in collision with fixed or stationary object, subsequent encounter |
| V80.82XS | Occupant of animal-drawn vehicle injured in collision with fixed or stationary object, sequela |
| V80.9 | Animal-rider or occupant of animal-drawn vehicle injured in other and unspecified transport accidents |
| V80.91 | Animal-rider injured in other and unspecified transport accidents |
| V80.910 | Animal-rider injured in transport accident with military vehicle |
| V80.910A | Animal-rider injured in transport accident with military vehicle, initial encounter |
| V80.910D | Animal-rider injured in transport accident with military vehicle, subsequent encounter |
| V80.910S | Animal-rider injured in transport accident with military vehicle, sequela |
| V80.918 | Animal-rider injured in other transport accident |
| V80.918A | Animal-rider injured in other transport accident, initial encounter |
| V80.918D | Animal-rider injured in other transport accident, subsequent encounter |
| V80.918S | Animal-rider injured in other transport accident, sequela |
| V80.919 | Animal-rider injured in unspecified transport accident |
| V80.919A | Animal-rider injured in unspecified transport accident, initial encounter |
| V80.919D | Animal-rider injured in unspecified transport accident, subsequent encounter |
| V80.919S | Animal-rider injured in unspecified transport accident, sequela |
| V80.92 | Occupant of animal-drawn vehicle injured in other and unspecified transport accidents |
| V80.920 | Occupant of animal-drawn vehicle injured in transport accident with military vehicle |
| V80.920A | Occupant of animal-drawn vehicle injured in transport accident with military vehicle, initial encounter |
| V80.920D | Occupant of animal-drawn vehicle injured in transport accident with military vehicle, subsequent encounter |
| V80.920S | Occupant of animal-drawn vehicle injured in transport accident with military vehicle, sequela |
| V80.928 | Occupant of animal-drawn vehicle injured in other transport accident |
| V80.928A | Occupant of animal-drawn vehicle injured in other transport accident, initial encounter |
| V80.928D | Occupant of animal-drawn vehicle injured in other transport accident, subsequent encounter |
| V80.928S | Occupant of animal-drawn vehicle injured in other transport accident, sequela |
| V80.929 | Occupant of animal-drawn vehicle injured in unspecified transport accident |
| V80.929A | Occupant of animal-drawn vehicle injured in unspecified transport accident, initial encounter |
| V80.929D | Occupant of animal-drawn vehicle injured in unspecified transport accident, subsequent encounter |
| V80.929S | Occupant of animal-drawn vehicle injured in unspecified transport accident, sequela |
| V82.8 | Occupant of streetcar injured in other specified transport accidents |
| V82.8XXA | Occupant of streetcar injured in other specified transport accidents, initial encounter |
| V82.8XXD | Occupant of streetcar injured in other specified transport accidents, subsequent encounter |
| V82.8XXS | Occupant of streetcar injured in other specified transport accidents, sequela |
| V82.9 | Occupant of streetcar injured in unspecified traffic accident |
| V82.9XXA | Occupant of streetcar injured in unspecified traffic accident, initial encounter |
| V82.9XXD | Occupant of streetcar injured in unspecified traffic accident, subsequent encounter |
| V82.9XXS | Occupant of streetcar injured in unspecified traffic accident, sequela |
| V84 | Occupant of special vehicle mainly used in agriculture injured in transport accident |
| V84.0 | Driver of special agricultural vehicle injured in traffic accident |
| V84.0XXA | Driver of special agricultural vehicle injured in traffic accident, initial encounter |
| V84.0XXD | Driver of special agricultural vehicle injured in traffic accident, subsequent encounter |
| V84.0XXS | Driver of special agricultural vehicle injured in traffic accident, sequela |
| V84.1 | Passenger of special agricultural vehicle injured in traffic accident |
| V84.1XXA | Passenger of special agricultural vehicle injured in traffic accident, initial encounter |
| V84.1XXD | Passenger of special agricultural vehicle injured in traffic accident, subsequent encounter |
| V84.1XXS | Passenger of special agricultural vehicle injured in traffic accident, sequela |
| V84.2 | Person on outside of special agricultural vehicle injured in traffic accident |
| V84.2XXA | Person on outside of special agricultural vehicle injured in traffic accident, initial encounter |
| V84.2XXD | Person on outside of special agricultural vehicle injured in traffic accident, subsequent encounter |
| V84.2XXS | Person on outside of special agricultural vehicle injured in traffic accident, sequela |
| V84.3 | Unspecified occupant of special agricultural vehicle injured in traffic accident |
| V84.3XXA | Unspecified occupant of special agricultural vehicle injured in traffic accident, initial encounter |
| V84.3XXD | Unspecified occupant of special agricultural vehicle injured in traffic accident, subsequent encounter |
| V84.3XXS | Unspecified occupant of special agricultural vehicle injured in traffic accident, sequela |
| V84.4 | Person injured while boarding or alighting from special agricultural vehicle |
| V84.4XXA | Person injured while boarding or alighting from special agricultural vehicle, initial encounter |
| V84.4XXD | Person injured while boarding or alighting from special agricultural vehicle, subsequent encounter |
| V84.4XXS | Person injured while boarding or alighting from special agricultural vehicle, sequela |
| V84.5 | Driver of special agricultural vehicle injured in nontraffic accident |
| V84.5XXA | Driver of special agricultural vehicle injured in nontraffic accident, initial encounter |
| V84.5XXD | Driver of special agricultural vehicle injured in nontraffic accident, subsequent encounter |
| V84.5XXS | Driver of special agricultural vehicle injured in nontraffic accident, sequela |
| V84.6 | Passenger of special agricultural vehicle injured in nontraffic accident |
| V84.6XXA | Passenger of special agricultural vehicle injured in nontraffic accident, initial encounter |
| V84.6XXD | Passenger of special agricultural vehicle injured in nontraffic accident, subsequent encounter |
| V84.6XXS | Passenger of special agricultural vehicle injured in nontraffic accident, sequela |
| V84.7 | Person on outside of special agricultural vehicle injured in nontraffic accident |
| V84.7XXA | Person on outside of special agricultural vehicle injured in nontraffic accident, initial encounter |
| V84.7XXD | Person on outside of special agricultural vehicle injured in nontraffic accident, subsequent encounter |
| V84.7XXS | Person on outside of special agricultural vehicle injured in nontraffic accident, sequela |
| V84.9 | Unspecified occupant of special agricultural vehicle injured in nontraffic accident |
| V84.9XXA | Unspecified occupant of special agricultural vehicle injured in nontraffic accident, initial encounter |
| V84.9XXD | Unspecified occupant of special agricultural vehicle injured in nontraffic accident, subsequent encounter |
| V84.9XXS | Unspecified occupant of special agricultural vehicle injured in nontraffic accident, sequela |
| V86.59 | Driver of other special all-terrain or other off-road motor vehicle injured in nontraffic accident |
| V86.59XA | Driver of other special all-terrain or other off-road motor vehicle injured in nontraffic accident, initial encounter |
| V86.59XD | Driver of other special all-terrain or other off-road motor vehicle injured in nontraffic accident, subsequent encounter |
| V86.59XS | Driver of other special all-terrain or other off-road motor vehicle injured in nontraffic accident, sequela |
| V86.69 | Passenger of other special all-terrain or other off-road motor vehicle injured in nontraffic accident |
| V86.69XA | Passenger of other special all-terrain or other off-road motor vehicle injured in nontraffic accident, initial encounter |
| V86.69XD | Passenger of other special all-terrain or other off-road motor vehicle injured in nontraffic accident, subsequent encounter |
| V86.69XS | Passenger of other special all-terrain or other off-road motor vehicle injured in nontraffic accident, sequela |
| V86.79 | Person on outside of other special all-terrain or other off-road motor vehicles injured in nontraffic accident |
| V86.79XA | Person on outside of other special all-terrain or other off-road motor vehicles injured in nontraffic accident, initial encounter |
| V86.79XD | Person on outside of other special all-terrain or other off-road motor vehicles injured in nontraffic accident, subsequent encounter |
| V86.79XS | Person on outside of other special all-terrain or other off-road motor vehicles injured in nontraffic accident, sequela |
| V86.99 | Unspecified occupant of other special all-terrain or other off-road motor vehicle injured in nontraffic accident |
| V86.99XA | Unspecified occupant of other special all-terrain or other off-road motor vehicle injured in nontraffic accident, initial encounter |
| V86.99XD | Unspecified occupant of other special all-terrain or other off-road motor vehicle injured in nontraffic accident, subsequent encounter |
| V86.99XS | Unspecified occupant of other special all-terrain or other off-road motor vehicle injured in nontraffic accident, sequela |
| V88.9 | Person injured in other specified (collision)(noncollision) transport accidents involving nonmotor vehicle, nontraffic |
| V88.9XXA | Person injured in other specified (collision)(noncollision) transport accidents involving nonmotor vehicle, nontraffic, initial encounter |
| V88.9XXD | Person injured in other specified (collision)(noncollision) transport accidents involving nonmotor vehicle, nontraffic, subsequent encounter |
| V88.9XXS | Person injured in other specified (collision)(noncollision) transport accidents involving nonmotor vehicle, nontraffic, sequela |
| W30 | Contact with agricultural machinery |
| W30.0 | Contact with combine harvester |
| W30.0XXA | Contact with combine harvester, initial encounter |
| W30.0XXD | Contact with combine harvester, subsequent encounter |
| W30.0XXS | Contact with combine harvester, sequela |
| W30.1 | Contact with power take-off devices (PTO) |
| W30.1XXA | Contact with power take-off devices (PTO), initial encounter |
| W30.1XXD | Contact with power take-off devices (PTO), subsequent encounter |
| W30.1XXS | Contact with power take-off devices (PTO), sequela |
| W30.2 | Contact with hay derrick |
| W30.2XXA | Contact with hay derrick, initial encounter |
| W30.2XXD | Contact with hay derrick, subsequent encounter |
| W30.2XXS | Contact with hay derrick, sequela |
| W30.3 | Contact with grain storage elevator |
| W30.3XXA | Contact with grain storage elevator, initial encounter |
| W30.3XXD | Contact with grain storage elevator, subsequent encounter |
| W30.3XXS | Contact with grain storage elevator, sequela |
| W30.8 | Contact with other specified agricultural machinery |
| W30.81 | Contact with agricultural transport vehicle in stationary use |
| W30.81XA | Contact with agricultural transport vehicle in stationary use, initial encounter |
| W30.81XD | Contact with agricultural transport vehicle in stationary use, subsequent encounter |
| W30.81XS | Contact with agricultural transport vehicle in stationary use, sequela |
| W30.89 | Contact with other specified agricultural machinery |
| W30.89XA | Contact with other specified agricultural machinery, initial encounter |
| W30.89XD | Contact with other specified agricultural machinery, subsequent encounter |
| W30.89XS | Contact with other specified agricultural machinery, sequela |
| W30.9 | Contact with unspecified agricultural machinery |
| W30.9XXA | Contact with unspecified agricultural machinery, initial encounter |
| W30.9XXD | Contact with unspecified agricultural machinery, subsequent encounter |
| W30.9XXS | Contact with unspecified agricultural machinery, sequela |
| W55.1 | Contact with horse |
| W55.11 | Bitten by horse |
| W55.11XA | Bitten by horse, initial encounter |
| W55.11XD | Bitten by horse, subsequent encounter |
| W55.11XS | Bitten by horse, sequela |
| W55.12 | Struck by horse |
| W55.12XA | Struck by horse, initial encounter |
| W55.12XD | Struck by horse, subsequent encounter |
| W55.12XS | Struck by horse, sequela |
| W55.19 | Other contact with horse |
| W55.19XA | Other contact with horse, initial encounter |
| W55.19XD | Other contact with horse, subsequent encounter |
| W55.19XS | Other contact with horse, sequela |
| W55.2 | Contact with cow |
| W55.21 | Bitten by cow |
| W55.21XA | Bitten by cow, initial encounter |
| W55.21XD | Bitten by cow, subsequent encounter |
| W55.21XS | Bitten by cow, sequela |
| W55.22 | Struck by cow |
| W55.22XA | Struck by cow, initial encounter |
| W55.22XD | Struck by cow, subsequent encounter |
| W55.22XS | Struck by cow, sequela |
| W55.29 | Other contact with cow |
| W55.29XA | Other contact with cow, initial encounter |
| W55.29XD | Other contact with cow, subsequent encounter |
| W55.29XS | Other contact with cow, sequela |
| W55.3 | Contact with other hoof stock |
| W55.31 | Bitten by other hoof stock |
| W55.31XA | Bitten by other hoof stock, initial encounter |
| W55.31XD | Bitten by other hoof stock, subsequent encounter |
| W55.31XS | Bitten by other hoof stock, sequela |
| W55.32 | Struck by other hoof stock |
| W55.32XA | Struck by other hoof stock, initial encounter |
| W55.32XD | Struck by other hoof stock, subsequent encounter |
| W55.32XS | Struck by other hoof stock, sequela |
| W55.39 | Other contact with other hoof stock |
| W55.39XA | Other contact with other hoof stock, initial encounter |
| W55.39XD | Other contact with other hoof stock, subsequent encounter |
| W55.39XS | Other contact with other hoof stock, sequela |
| W55.4 | Contact with pig |
| W55.41 | Bitten by pig |
| W55.41XA | Bitten by pig, initial encounter |
| W55.41XD | Bitten by pig, subsequent encounter |
| W55.41XS | Bitten by pig, sequela |
| W55.42 | Struck by pig |
| W55.42XA | Struck by pig, initial encounter |
| W55.42XD | Struck by pig, subsequent encounter |
| W55.42XS | Struck by pig, sequela |
| W55.49 | Other contact with pig |
| W55.49XA | Other contact with pig, initial encounter |
| W55.49XD | Other contact with pig, subsequent encounter |
| W55.49XS | Other contact with pig, sequela |
| W55.8 | Contact with other mammals |
| W55.81 | Bitten by other mammals |
| W55.81XA | Bitten by other mammals, initial encounter |
| W55.81XD | Bitten by other mammals, subsequent encounter |
| W55.81XS | Bitten by other mammals, sequela |
| W55.82 | Struck by other mammals |
| W55.82XA | Struck by other mammals, initial encounter |
| W55.82XD | Struck by other mammals, subsequent encounter |
| W55.82XS | Struck by other mammals, sequela |
| W55.89 | Other contact with other mammals |
| W55.89XA | Other contact with other mammals, initial encounter |
| W55.89XD | Other contact with other mammals, subsequent encounter |
| W55.89XS | Other contact with other mammals, sequela |
| W61.3 | Contact with chicken |
| W61.32 | Struck by chicken |
| W61.32XA | Struck by chicken, initial encounter |
| W61.32XD | Struck by chicken, subsequent encounter |
| W61.32XS | Struck by chicken, sequela |
| W61.33 | Pecked by chicken |
| W61.33XA | Pecked by chicken, initial encounter |
| W61.33XD | Pecked by chicken, subsequent encounter |
| W61.33XS | Pecked by chicken, sequela |
| W61.39 | Other contact with chicken |
| W61.39XA | Other contact with chicken, initial encounter |
| W61.39XD | Other contact with chicken, subsequent encounter |
| W61.39XS | Other contact with chicken, sequela |
| W61.4 | Contact with turkey |
| W61.42 | Struck by turkey |
| W61.42XA | Struck by turkey, initial encounter |
| W61.42XD | Struck by turkey, subsequent encounter |
| W61.42XS | Struck by turkey, sequela |
| W61.43 | Pecked by turkey |
| W61.43XA | Pecked by turkey, initial encounter |
| W61.43XD | Pecked by turkey, subsequent encounter |
| W61.43XS | Pecked by turkey, sequela |
| W61.49 | Other contact with turkey |
| W61.49XA | Other contact with turkey, initial encounter |
| W61.49XD | Other contact with turkey, subsequent encounter |
| W61.49XS | Other contact with turkey, sequela |
| W61.5 | Contact with goose |
| W61.51 | Bitten by goose |
| W61.51XA | Bitten by goose, initial encounter |
| W61.51XD | Bitten by goose, subsequent encounter |
| W61.51XS | Bitten by goose, sequela |
| W61.52 | Struck by goose |
| W61.52XA | Struck by goose, initial encounter |
| W61.52XD | Struck by goose, subsequent encounter |
| W61.52XS | Struck by goose, sequela |
| W61.59 | Other contact with goose |
| W61.59XA | Other contact with goose, initial encounter |
| W61.59XD | Other contact with goose, subsequent encounter |
| W61.59XS | Other contact with goose, sequela |
| W61.6 | Contact with duck |
| W61.61 | Bitten by duck |
| W61.61XA | Bitten by duck, initial encounter |
| W61.61XD | Bitten by duck, subsequent encounter |
| W61.61XS | Bitten by duck, sequela |
| W61.62 | Struck by duck |
| W61.62XA | Struck by duck, initial encounter |
| W61.62XD | Struck by duck, subsequent encounter |
| W61.62XS | Struck by duck, sequela |
| W61.69 | Other contact with duck |
| W61.69XA | Other contact with duck, initial encounter |
| W61.69XD | Other contact with duck, subsequent encounter |
| W61.69XS | Other contact with duck, sequela |
| Y92.7 | Farm as the place of occurrence of the external cause |
| Y92.71 | Barn as the place of occurrence of the external cause |
| Y92.72 | Chicken coop as the place of occurrence of the external cause |
| Y92.73 | Farm field as the place of occurrence of the external cause |
| Y92.74 | Orchard as the place of occurrence of the external cause |
| Y92.79 | Other farm location as the place of occurrence of the external cause |
| Y93.K2 | Activity, milking an animal |
| Y93.K3 | Activity, grooming and shearing an animal |
| Y93.K9 | Activity, other involving animal care |
